# Retraction

**DOI:** 10.2174/1874120701812010091

**Published:** 2018-09-11

The Publisher and Editor have retracted these articles [1 - 9] in accordance with good ethical practices. After thorough investigations we believe that the peer review process was compromised.

1. Wang Yanhua, Wu Fuhua, Guo Zhaohan, Peng Mingxing, Zhang Yanan, Pang Zhen Ling, Du Minhua, Zhang Caiying, and Liang Zian, “Optimization of Extraction Process for Polysaccharide in Salvia Miltiorrhiza Bunge Using Response Surface Methodology”, *Open Biomed. Eng. J.*, vol. 8, pp. 153-159, 2014.
2. Dapeng Wang, and Lan Zhang, “Fuzzy Analytic Hierarchy Process-based Chinese Resident Best Fitness Behavior Method Research”, *Open Biomed. Eng. J.*, vol. 9, pp. 271-275, 2015.
3. Zhang Mengyan, “Protection of Lotus Seedpod Proanthocyanidins on Organs and Tissues under High-intensity Excercise”, *Open Biomed. Eng. J.*, vol. 9, pp. 296-300, 2015.
4. Dongmei Li, “The Research on the Effect of the Food with Different Glycaemic Index and Glycaemic Load on the Immunity of Endurance Athletes”, *Open Biomed. Eng. J.*, vol. 9, pp. 305-309, 2015.
5. Fu Qiang, “Effect of Malate-oligosaccharide Solution on Antioxidant Capacity of Endurance Athletes”, *Open Biomed. Eng. J.*, vol. 9, pp. 326-329, 2015.
6. Sun Ronghui, “The Reasearch on the Anti-Fatigue Effect of Whey Protein Powder in Basketball Training”, *Open Biomed. Eng. J.*, vol. 9, pp. 330-334, 2015.
7. Wang Yanhua, Wu Fuhua, Guo Zhaohan, Peng Mingxing, Zhang Yanan, Pang Zhen Ling, Du Minhua, Zhang Caiying, and Liang Zian, “Optimization of Extraction Process for Polysaccharide in Salvia Miltiorrhiza Bunge Using Response Surface Methodology”, *Open Biomed. Eng. J.*, vol. 9, pp. 347-353, 2015.
8. Yong-Gan Zhang, Xue-Li Guo, Yan Song, Chao-Feng Miao, Chuang Zhang, and Ning-Heng Chen, “Diagnosis and Treatment of Vascular Surgery Related Infection”, *Open Biomed. Eng. J.*, vol. 9, pp. 250-255, 2015
9. Xianchun Wen, and Liling Yue, “The Influence of Corn Silk Polysaccharide on Signal Pathway of TGF-β1 in Type 2 Diabetic Mellitus Rat”, *Open Biomed. Eng. J.*, vol. 9, pp. 204-208, 2015.

